# Effects of combined exposure to heavy metals on lower respiratory flora and its role of lung injury in rats

**DOI:** 10.3389/fmicb.2025.1615130

**Published:** 2025-06-13

**Authors:** Ping Ding, Xiaoxia Wang, Nan Li, Shuxia Yu, Yiwen Zhang, Junpu Yu, Tian Tian, Rentong Chen, Bin Luo, Li Ma, Rongxuan Zhang

**Affiliations:** ^1^School of Public Health, Lanzhou University, Lanzhou, China; ^2^Department of Medical Administration, Gansu Provincial Maternity and Child-Care Hospital, Lanzhou, China; ^3^Department of Respiratory and Critical Care Medicine, The Second Hospital of Lanzhou City, Lanzhou, China

**Keywords:** heavy metal, exposure, respiratory microbiota, microbial dysbiosis, 16S rDNA sequencing, host-microbiota interaction

## Abstract

**Introduction:**

Exposure to heavy metals is a growing environmental concern. Although exposure to individual metals is associated with pulmonary damage, real-world exposure typically involves multiple metals simultaneously. This study hypothesizes that combined exposure to nickel, copper, and arsenic induces lung injury through disruption of the bronchoalveolar lavage fluid (BALF) microbial ecosystem in rats. The primary objective of this study was to verify the hypothesis.

**Methods:**

Thirty-two male Sprague–Dawley (SD) rats were randomly assigned to four groups: one control group and three exposure groups (low, medium, and high doses). The exposed groups received mixed heavy metal aerosols containing nickel (Ni), copper (Cu), and arsenic (As) at low (Ni: 210.9 ng/m^3^, Cu: 108.4 ng/m^3^, As: 104.6 ng/m^3^), medium (5 × low), and high (10 × low) concentrations. Exposure occurred via inhalation twice daily for 1 h over 90 days. Lung function was assessed non-invasively, and histological examinations, 16S ribosomal DNA (16S rDNA) sequencing, and microbial functional predictions were performed to evaluate exposure effects. We measured heavy metal concentrations in lung tissues and assessed the associations with microbial changes. Microbial community structure and function were further analyzed using LEfSe, PICRUSt2, and ecological network analysis.

**Results:**

Compared exposure to Ni, Cu, and As induced dose-dependent lung damage, including inflammation, alveolar deformation, and bronchial thickening, accompanied by significant declines in lung function, including a 21.2% reduction in tidal volume and a 34.5% increase in airway resistance in the high-dose group (*P* < 0.05). Microbial diversity and phylogenetic richness were significantly reduced (Chao1, Richness, ACE, *P* < 0.05), with taxonomic shifts characterized by the enrichment of metal-resistant genera (*Pseudomonas, Burkholderia*) and depletion of sensitive taxa (*Ralstonia, Achromobacter*). Functional prediction suggested impairments in xenobiotic metabolism and amino acid biosynthesis. Ecological network complexity declined with increasing exposure dose. Microbiota dysbiosis is strongly associated with lung function impairments.

**Conclusions:**

Combined exposure to Ni, Cu, and As disrupts respiratory microbiota and impairs lung function in rats. These findings highlight a critical link between environmental heavy metal exposure and respiratory health, underscoring the need for stricter regulation of metal pollutants and further research into microbiota-related lung injury mechanisms.

## 1 Introduction

Chronic respiratory disease refers to a group of conditions that persistently impair respiratory system function, including chronic obstructive pulmonary disease (COPD), asthma, and others. These diseases are characterized by a prolonged course, slow recovery, and frequent relapse (Lin et al., 2018), representing a major global health burden. Lung function, a key indicator for assessing chronic lung disease, is widely used to evaluate the impact of environmental pollution, particularly to quantify the effects of air pollution on the respiratory tract (Duprez and Jacobs, 2018; Zheng et al., 2023).

Heavy metals are commonly defined as elements with an atomic number exceeding 20 and a density >5 g/cm^3^, including metalloids like arsenic (As) due to their similar chemical properties and environmental behavior (Tchounwou et al., 2012; Gautam et al., 2016; Jacob et al., 2018). In recent years, intensified industrial activities, fossil fuel combustion, and heavy fertilizer use have led to rising levels of heavy metals in the environment (Long et al., 2021; Lavanya et al., 2024). This issue is particularly pronounced in China and other developing countries (Jacob et al., 2018; Liu et al., 2022). Inhaled heavy metal particles typically deposited in the bronchioles and alveoli, causing severe lung damage. Moreover, these pollutants may enter the blood circulation through pathways such as ingestion or absorption through the skin, triggering systemic inflammation that further exacerbates lung damage (Balali-Mood et al., 2021; Hashem et al., 2021).

Although numerous studies have confirmed the adverse effects of heavy metal exposure on lung function, they have predominantly focused on individual metals and mechanisms such as inflammation, oxidative stress, and apoptosis (Wu et al., 2022), or on the gastrointestinal microbiota under metal exposure (Li X. et al., 2019). However, real-world exposure involves complex metal mixtures, and their combined effects, particularly on the lower respiratory tract microbiome, a key interface for immune defense and pollutant interaction, remain poorly understood (Xue et al., 2020).

Historically, healthy lungs were thought to be sterile environments (Chambers et al., 2014; Taylor et al., 2015). However, recent studies have revealed a complex microbial ecosystem within the airways and lung tissues that serves as a “gatekeeper” of respiratory health (Morris et al., 2013; Venkataraman et al., 2015). Imbalances in these microbiota are associated with various respiratory diseases, including COPD, asthma, and cystic fibrosis (Huang et al., 2015; Li et al., 2017), with changes in microbial communities correlating with reduced lung function (Wang et al., 2019; Zhang et al., 2023). However, research on microbiome changes induced by heavy metal exposure has primarily focused on the gut flora, with limited studies exploring the interaction between respiratory microbiota and heavy metal exposure.

Based on the above, we hypothesize that combined exposure to nickel, copper, and arsenic induces dose-dependent lung injury through disruption of the respiratory microbiota. Such exposure is expected to alter the composition and function of airway microbial communities, leading to reduced microbial diversity and the enrichment of pollutant-tolerant taxa. These microbial shifts may contribute to or mediate the observed decline in lung function, suggesting a potential link between microbiota dysbiosis and metal-induced pulmonary toxicity.

In this study, we evaluated lung structure and functional parameters and profiled the respiratory microbiota in rats exposed to varying concentrations of mixed heavy metal aerosols. The objective was to investigate the impact of combined heavy metal exposure on pulmonary injury and to elucidate the potential role of BALF microbiota dysbiosis in this process. This study will provide a novel perspective on pollution-induced respiratory dysfunction and support future efforts toward microbiota-informed environmental health policies.

## 2 Materials and methods

### 2.1 Animal exposure programs

Our research team has long focused on the health impacts of heavy metal pollution in an industrial region northwest of China. Based on our atmospheric monitoring data (Table 1), nickel (Ni), copper (Cu), and arsenic (As) were identified as the predominant metals in atmospheric PM_2.5_ from the industrial zone, with average concentrations of 210.90 ± 18.70 ng/m^3^ for Ni, 108.35 ± 8.21 ng/m^3^ for Cu, and 104.60 ± 8.38 ng/m^3^ for As. These values were significantly higher than those observed in a nearby non-industrial area (3.24 ± 0.34 ng/m^3^ for Ni, 4.67 ± 0.31 ng/m^3^ for Cu, and 7.61 ± 0.66 ng/m^3^ for As) (*P* < 0.01).

**Table 1:** The PM_2.5_ concentration and metal content in ambient air in the study area.

**Composition**	**Industrial area**	**Non-industrial area**	***P***
*PM_2.5_ (μg/m^3^)*	44.67 ± 2.92	46.80 ± 3.08	>0.05
*Ni (ng/m^3^)*	210.90 ± 18.70	3.24 ± 0.34	<0.01
*Cu (ng/m^3^)*	108.35 ± 8.21	4.67 ± 0.31	<0.01
*As (ng/m^3^)*	104.60 ± 8.38	7.61 ± 0.66	<0.01

Data are presented as mean ± standard error (SE). Statistical comparisons between contaminated and control areas were performed using two-sample *t*-tests.

Based on these findings, we designed three exposure groups, using the concentrations of Ni, Cu, and As detected in the ambient PM_2.5_ of the industrial region as the reference for the low-dose group (Table 1). The medium and high doses were set at 5 and 10 times the low-dose level, respectively. To replicate environmental exposure conditions, an aerosol mixture was prepared using nickel sulfate hexahydrate (NiSO_4_·6H_2_O), copper sulfate pentahydrate (CuSO4·5H_2_O), and sodium arsenite (NaAsO_2_), dissolved in deionized water and aerosolized with a nebulizer for controlled inhalation delivery.

**Figure 1:**
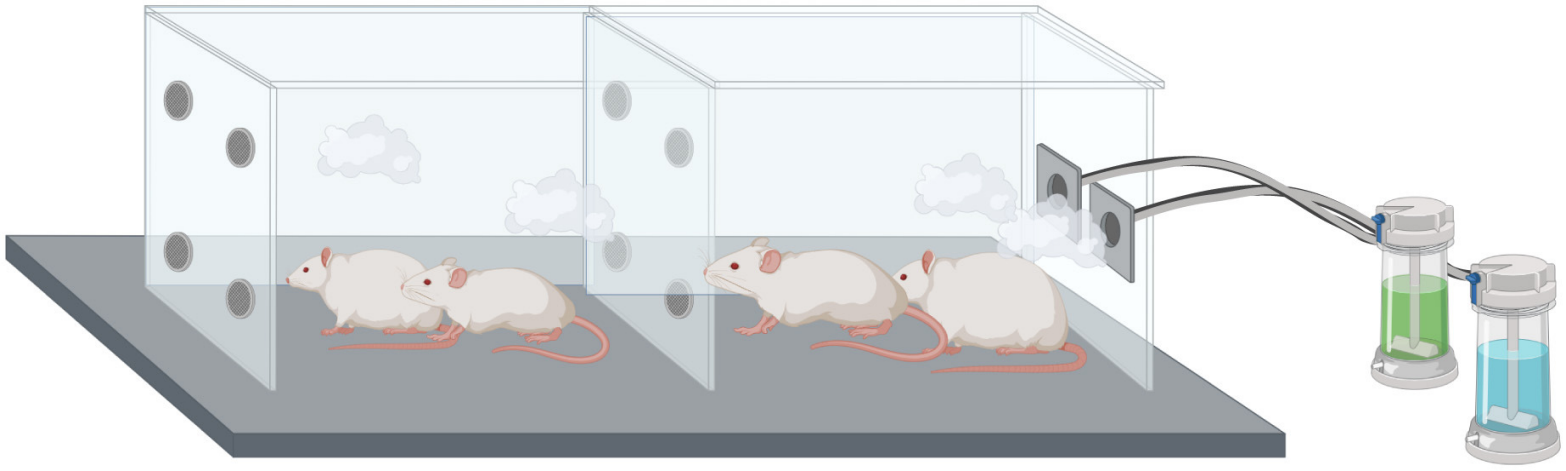
Schematic representation of aerosol exposure in mice.

Thirty-two SPF-grade SD male rats, aged 6–8 weeks and weighing between 180–220 g, were purchased from the Laboratory Animal Center of Lanzhou University. The rats were randomly assigned to four groups (*n* = 8 per group): control group (Group C), low-dose group (Group L), medium-dose group (Group M), and high-dose group (Group H). Group C was housed in a conventional laboratory environment without aerosol atomization exposure, while Groups L, M, and H were respectively placed in isolated chambers within a transparent exposure box for aerosol exposure to solutions of varying concentration gradients (Figure 1). The transparent exposure box measured 0.5 m × 0.3 m × 0.35 m (length × width × height), and contained a mesh fence to separate the box into isolated chambers to prevent rats from aggregating and burying their mouths and noses into each other's hairs (similar to “wearing a mask”), which would affect the effect of the exposure to the aerosol. The right side of the chamber was connected to a nebulizer serving as the inlet of the heavy metal aerosol mixture, while the left side was connected to a particle sampler to monitor the aerosol concentration in the exposure chamber.

The exposure was conducted twice daily, with each session lasting 1 h. After each exposure, the rats were returned to their cages. The exposure protocol spanned 90 days and was performed in a fume hood connected to handling and collection units. The ambient temperature was maintained at 18–26°C, and the relative humidity at 40%−70%.

### 2.2 Non-invasive lung function assessment in rats

Within 24 h of completing exposure, lung function was assessed non-invasively in all rats using the NAM system (DSI BUXCO, USA). Eight rats per group were individually placed in the cavity for 10 min to acclimate, after which relevant parameters were recorded, including respiratory rate (f), tidal volume (Tv), specific airway resistance (sRaw), airway resistance (Raw), maximal inspiratory flow rate (PIF), maximal expiratory flow rate (PEF), functional residual air volume (Frc), and expiratory flow rate at mid-expiration (EF50).

### 2.3 Collection of rat bronchoalveolar lavage fluid and lung tissue

Bronchoalveolar lavage fluid (BALF) was collected from one lung of each rat, with the contralateral lung harvested for tissue analysis. Rats were anesthetized with an intraperitoneal injection of 0.3 ml/100 g chloral hydrate, and the trachea and lungs were exposed. The epidermis of the animals was sterilized with 75% ethanol, and sterilized grade surgical instruments were used throughout. Under sterile conditions, the thoracic cavity was opened, and one lung was ligated at the bronchus for isolation. A tracheal cannula was inserted into each rat, and 10 ml of phosphate-buffered saline (PBS) was slowly injected into the lungs, with a dwell time of 30–60 seconds instillation. The lavage was repeated 2–3 times, and the recovered BALF was transferred to a sterile test tube, centrifuged to separate the cells and supernatant, and the supernatant was stored in a −80°C refrigerator for subsequent analysis. Concurrently, lung tissues were washed with saline and divided into two parts. One portion was fixed in 4% paraformaldehyde solution for hematoxylin and eosin (H&E) staining, while the other was snap-frozen in liquid nitrogen and stored at −80°C for heavy metal analysis.

### 2.4 Lung histopathology

Fixed rat lung tissues were dehydrated with alcohol and xylene, embedded in paraffin, and sectioned into 3–5 μm thin slices using a machine. The sections were stained with hematoxylin and eosin (H&E), coverslipped, and examined microscopically for histopathological analysis.

### 2.5 Determination of heavy metals in rat lung tissue

Heavy metal concentrations in lung tissue were determined using inductively coupled plasma mass spectrometry (ICP-MS) technique. Approximately 20–30 mg of lung tissue was digested in 10 ml of concentrated nitric acid using a microwave digestor for 1 h. After cooling and acid evaporation, the digestate was diluted, filtered, and analyzed for Ni, Cu, and As concentrations by ICP-MS.

### 2.6 16S rDNA sequencing of rat alveolar lavage fluid

Bacterial genomic DNA was extracted in BALF using the Mabiote Bacterial DNA Mini Kit, and the concentration and quality of the extracted genomic DNA were assessed with a Thermo Scientific Nanodrop One (Thermo Fisher Scientific, MA, USA). For each group, only BALF samples with qualified bacterial DNA were selected for 16S rRNA gene sequencing, while those with insufficient DNA quality or quantity were excluded to reduce contamination risks and ensure data reliability (Saladié et al., 2020; Baker et al., 2021). The V3-V4 region of 16S rDNA was amplified using the Illumina HiSeq platform with amplification primers 338F (5′-ACTCCTACGGGGAGGCAGCA- 3′) and 806R (5′-GGACTACHVGGGTWTCTAAT- 3′). Each amplification reaction consisted of denaturation at 98°C for 30 seconds, followed by 30 cycles of amplification (10 seconds at 98°C, annealing at 50°C for 30 seconds, and extension at 72°C for 1 min), concluding with a final extension at 72°C for 2 min. Amplicons were purified PCR amplicons from agarose gels and sequenced. Raw sequencing data were processed using QIIME2 (version 2020.11.0), including trimming of low-quality sequences and quality filtering with Cutadapt. Paired-end reads were assembled by FLASH 1.2.11. The Raw Tags sequence quality is filtered by using fastp (an ultra-fast all-in-one FASTQ preprocessor, version 0.14.1). Performs sliding window quality clipping (-W 4 -M 20) on Raw Tags data to obtain valid splicing fragments (Clean Tags).

For each sample, we used Deblur to process sequences, identify amplicon sequence variants (ASVs), and assign ASVs to taxonomic taxa (Amir et al., 2017).

### 2.7 Statistical analysis

We conducted dilution curve analysis and calculated multiple alpha diversity indices, including Chao1, ACE, Shannon_e, Simpson, Richness, Jost, dominance, Good's-coverage, and PD_whole_tree indices. The Kruskal-Wallis rank-sum test was applied to compare alpha diversity differences across groups.

Beta diversity analysis was employed to assess differences in species composition and structural organization among various biomes. A distance matrix was constructed to calculate the distances between biological communities for principal coordinate analysis (PCoA). β diversity was visualized using PCoA based on the ASV abundance table, utilizing the *vegan* package^1^ in R. The Bray-Curtis, weighted-UniFrac, and unweighted-UniFrac distance algorithms were applied.

Differences in bacterial community composition were assessed using PCoA at the ASV level, with Bray-Curtis, weighted and unweighted UniFrac distance metrics. Statistical significance was assessed by analysis of similarity (ANOSIM). Linear discriminant analysis of effect size (LEfSe) was applied to identify biomarkers that could lead to differences between the four groups, with LDA scores >3.5 as the threshold for distinguishing significantly different taxa. Concentration of Ni, Cu, and As in rat lung tissue was quantified, and their associations with lung function and respiratory microbial communities were investigated. To assess functional differences in metabolic pathways, microbial functional genes were predicted using the KEGG database.

All statistical analyses were performed using SPSS (version 26.0) and R (version 4.2.2). The Mantel test^2^ implemented in R was used to calculate correlation coefficients between the environmental factor matrix and community composition matrix, assessing significant correlations existed and the influence of environmental factors on community structure. Distance matrix calculations employed the Manhattan, Euclidean, and Bray-Curtis distance algorithms.

### 2.8 Microbial ecological networks analysis

Bacterial molecular ecological networks (MENs) in the BALF were constructed using the Integrated Network Analysis Pipeline (iNAP) (Feng et al., 2022), an online tool for ecological network analysis. Networks were inferred using the Meinschausen-Buhlmann (MB) neighborhood selection method, implemented through the SParse InversE Covariance Estimation (SPEIC-EASI) algorithm (Kurtz et al., 2015). The resulting networks were further explored and visualized with Gephi (Bastian et al., 2009), an interactive platform, utilizing the Fruchterman-Reingold layout, which provides an intuitive representation of network structures.

During network construction, only ASVs detected in >80% of samples were retained to ensure reliability and minimize noise. Key topological parameters and node scores were calculated using Gephi,^3^ including average degree (number of edges per node, reflecting connectivity), average path length (shortest distance between two nodes), network diameter (longest path between nodes), and clustering coefficient (degree of interconnection among a node's neighbors, indicating localized density). Community structure was identified by optimizing modularity, which quantified the strength of division into distinct clusters or modules. Networks with higher modularity exhibited dense intra-cluster connections and sparse inter-cluster connections, signifying distinct functional or ecological groupings.

Keystone taxa were identified based on Gephi-calculated parameters: degree >7, harmonic closeness centrality >0.28, and betweenness centrality <0.18 (Banerjee et al., 2019). The degree distribution was wide, with the highest degree (up to 18) representing <1% of nodes, and approximately 20% of nodes had degrees >7. Consequently, the degree threshold was adjusted from 10 to 7 to capture relevant taxa.

Microbial functions were predicted using DESeq2 for differential abundance analysis (McCarthy et al., 2012) and the Kyoto Encyclopaedia of Genes and Genomes (KEGG) database for metabolic pathway analysis (Kanehisa et al., 2017). KEGG enrichment analysis was performed using the *clusterProfiler* R package (Wu et al., 2021).

The correlation network map, correlation heat map, KEGG enrichment analysis and functional prediction volcano map among microbiota were generated using ChiPlot^4^ (accessed January 2025). For clarity, all abbreviations used in this manuscript are provided in Supplementary Table S1.

## 3 Results

### 3.1 General status and weight

Rats in Group L displayed normal behavior. Group M showed dull fur, reduced mobility, and occasional sneezing. Group H experienced fur thinning, nasal bleeding, respiratory distress, lethargy, and decreased food and water intake, worsening over time. Although all groups of rats gained weight (Table 2), the control group began to gain more weight than the other groups after 6 weeks of exposure, which may indicate that heavy metal exposure slowed down the rate of body weight gain. However, differences in body weight gain among groups were not statistically significant (*P* > 0.05).

**Table 2:** Change in body weight of rats in each group.

**Groups**	**Starting weight (g)**	**Final weight (g)**	**t**	***P***
C	190.5 ± 14.7	416.4 ± 22.7	22.36	<0.01
L	195.3 ± 8.7	391.8 ± 22.4	26.22	<0.01
M	190.8 ± 9.4	382.2 ± 32.3	20.65	<0.01
H	189.7 ± 7.9	373.6 ± 38.02	14.41	<0.01

Average weight in each group (*n* = 8). The T and *P* values are the values obtained after paired *t-*tests of the body weight of each group of mice before and after exposure. Data were shown as mean ± SE.

### 3.2 Levels of Ni, Cu, and As in rat lung tissue

As shown in Table 3, the concentrations of Ni and As in rat lung tissues exhibited significant increases with escalating exposure doses of the mixed aerosol, culminating in the highest levels in the H group (*P* < 0.01). In contrast, although the overall comparison across all groups did not reveal significant differences in Cu levels (*P* = 0.352), pairwise comparisons between each experimental group and the control group unveiled significant elevations in Cu concentrations in the experimental groups (*P* < 0.05). These results underscore that varying doses of Ni and As aerosol exposure significantly augment the accumulation of these heavy metals in rat lungs.

**Table 3:** Changes in heavy metal content in the lungs of rats in various groups (mg/kg).

**Metals**	**Group C**	**Group L**	**Group M**	**Group H**
Ni	0.17 ± 0.11	1.21 ± 0.75^*^	1.34 ± 0.10^#^	1.55 ± 0.72^*&^
Cu	1.05 ± 0.06	1.32 ± 0.54^*^	1.52 ± 0.53^*^	1.68 ± 0.57^*^
As	2.12 ± 0.28	2.19 ± 0.38^*^	3.94 ± 0.40^*#&^	4.55 ± 0.61^*#&^

^*^Represents statistically significant differences in the other three groups compared with group C. ^&^Stands for Group M and H with statistically significant differences compared with Group L. ^#^Represents a statistically significant difference between Group H and Group M (*P* < 0.05). Data were shown as mean ± SE.

### 3.3 Lung inflammation and lung function differences

**Figure 2:**
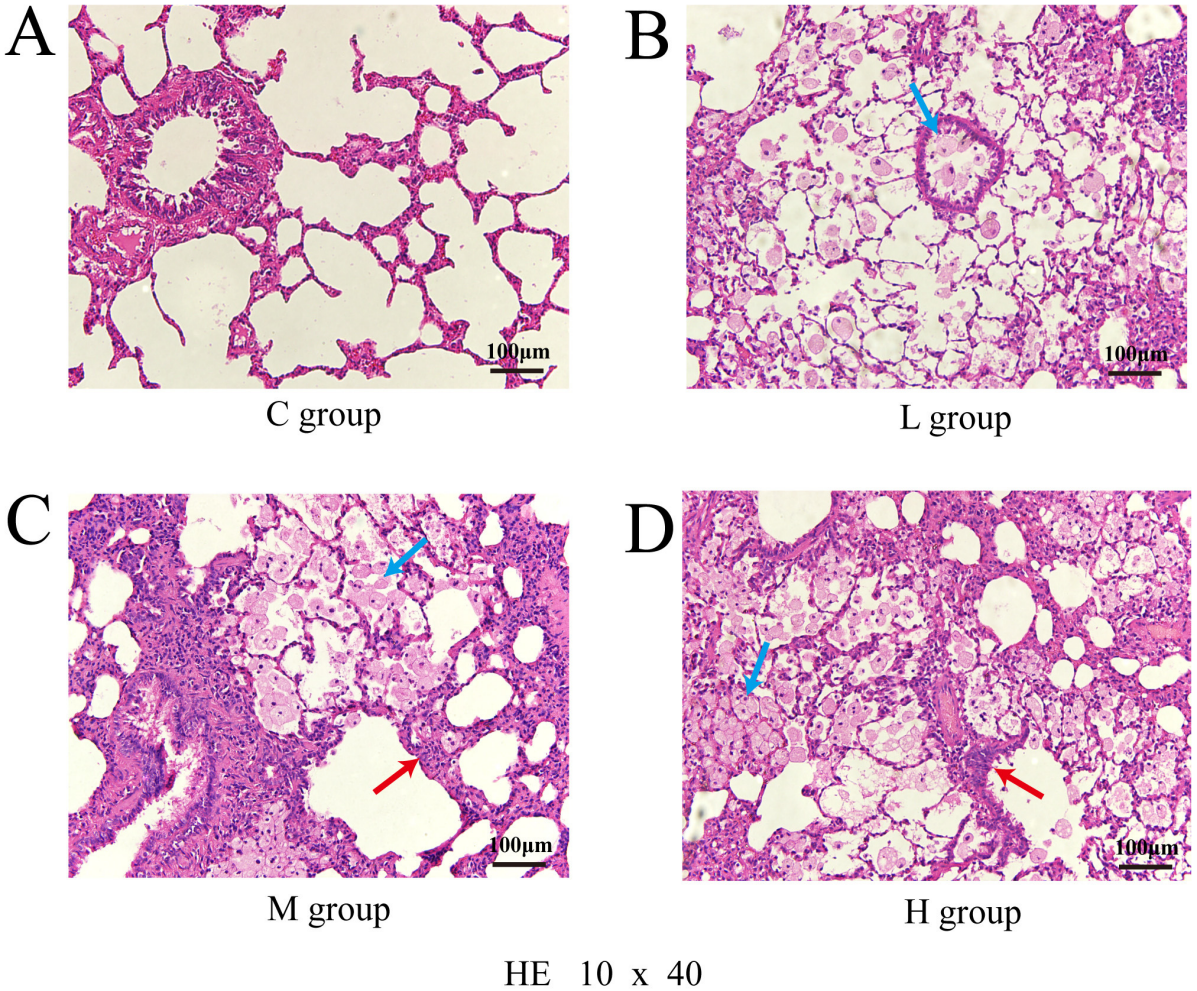
Pathological section of lung injury stained with hematoxylin and eosin (HE), observed under a light microscope at 10 × 40 magnification; Control group **(A)**; Low-dose group **(B)**; Medium-dose group **(C)**; High-dose group **(D)**; Blue arrows point to dust cells and red arrows to inflammatory cells.

In Group C, the respiratory tract and alveolar structures remained intact with no abnormal changes. Group L rats exhibited mild inflammation and vasodilation. Group M rats displayed enlarged alveoli, thickened bronchial walls, epithelial cell detachment, and moderate inflammation. Group H rats showed severe lung and bronchial damage, with disorganized alveolar and bronchial structures and abundant inflammatory cell infiltration. The number of dust cells increased progressively with higher exposure doses (Figure 2).

**Figure 3:**
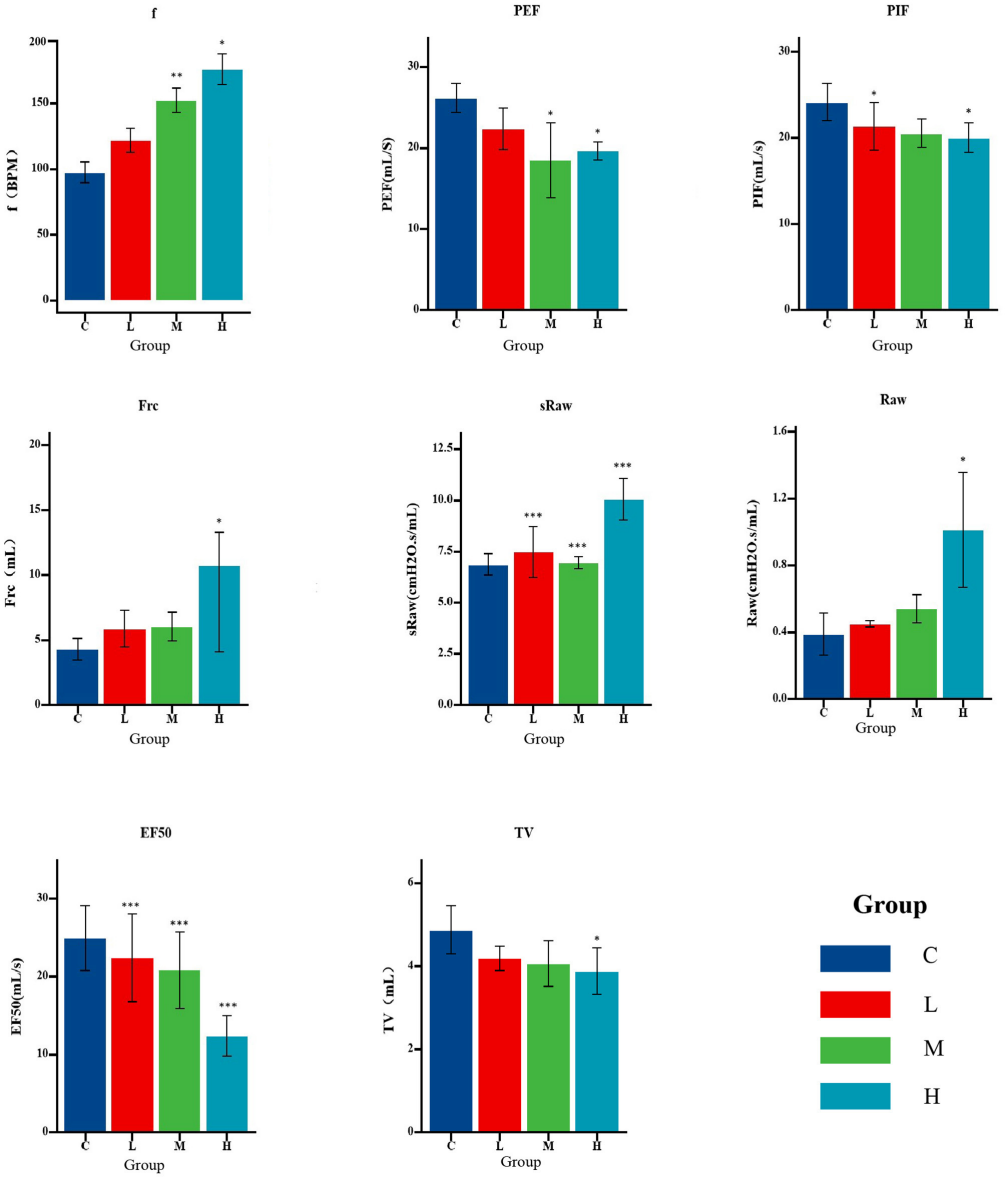
Pulmonary function parameters of rats in each exposure group (*n* = 8 per group). Bar graphs show mean ± standard error (SE) for each index. Statistical analysis was performed using a one-way ANOVA test; *P < 0.05, **P < 0.01, ***P < 0.001 compared to control (Group C).

With increasing heavy metal exposure, respiratory frequency (f), airway resistance (sRaw, Raw), and functional residual capacity (FRC) tended to increase, whereas tidal volume (Tv), peak inspiratory flow rate (PIF), peak expiratory flow rate (PEF), and flow rate at 50% expiration (EF50) decreased (Figure 3).

**Figure 4:**
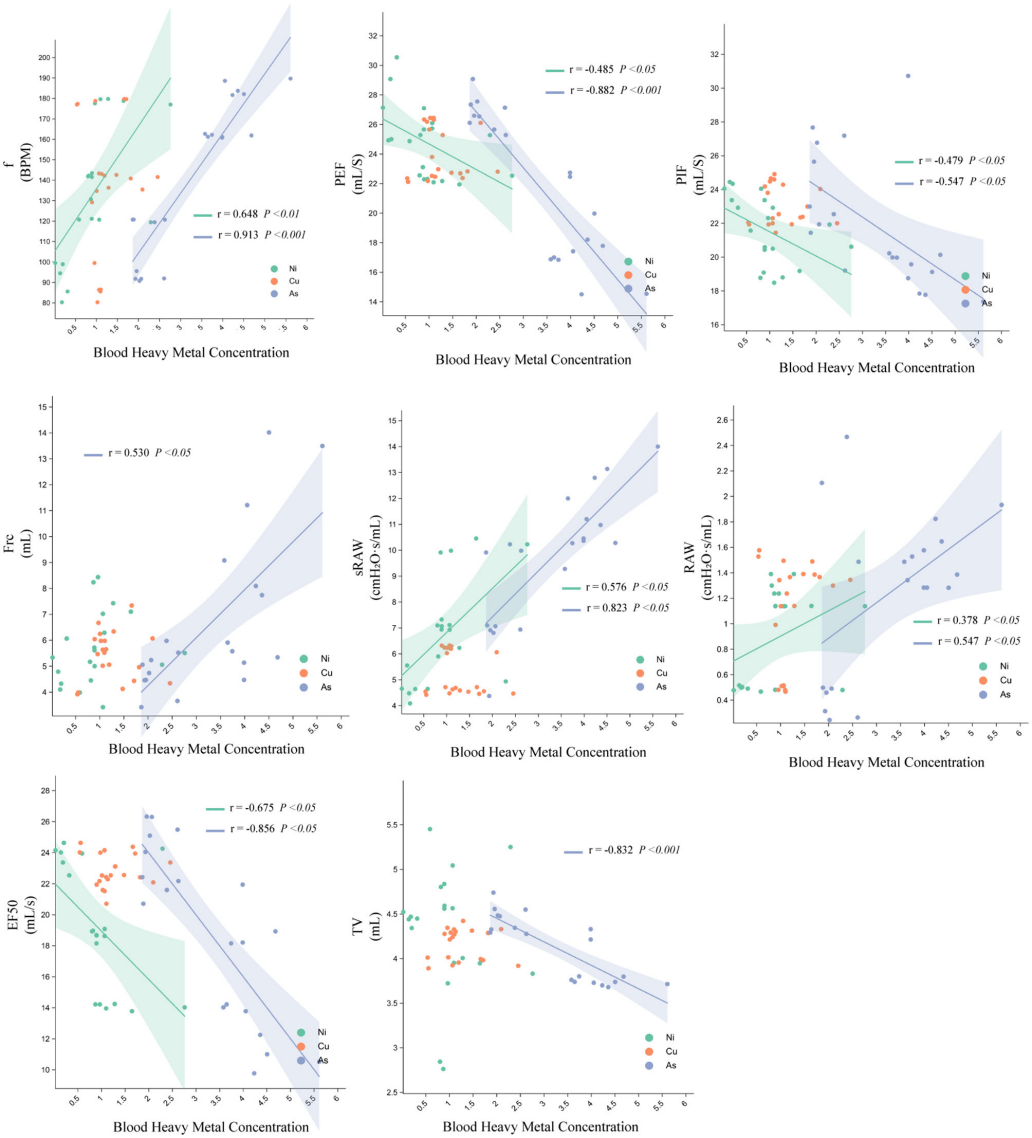
Relationship between Ni, Cu, and As heavy metal concentrations and pulmonary function parameters in rats.

FRC was positively correlated with lung arsenic (As) concentration, and f, sRaw, and Raw were positively correlated with Ni and As concentrations. Tv was negatively correlated with As concentration, and PIF, PEF, and EF50 were negatively correlated with Ni and As concentrations. The correlation between As and lung function was stronger than that with Ni, while copper (Cu) concentrations showed no significant correlations with lung function indices (Figure 4).

### 3.4 Bacterial population structure in alveolar lavage fluid of rats

**Figure 5:**
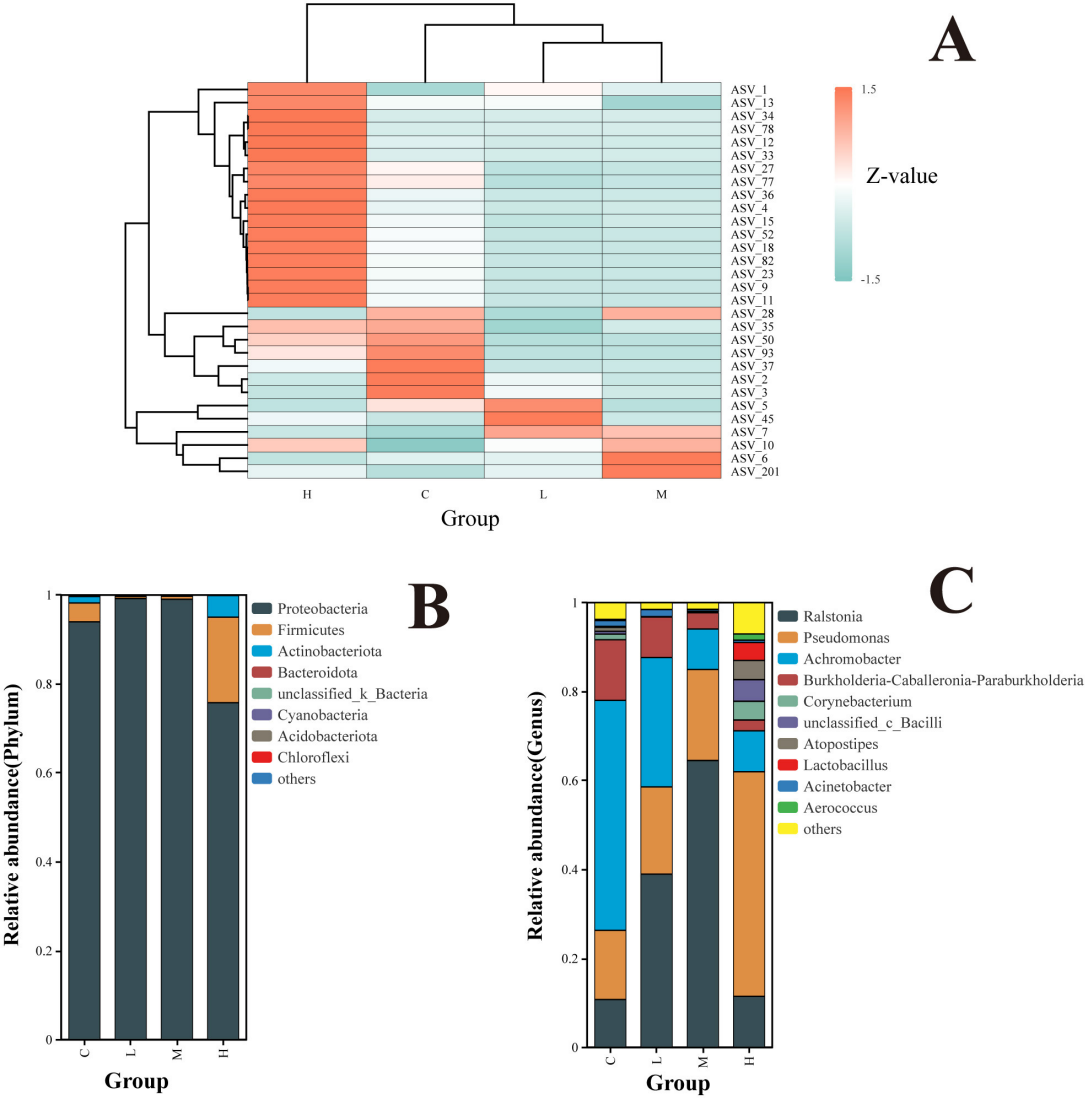
Clustering of species relative abundance at the ASV level **(A)**. Z-scores represent species abundance deviations from the mean across samples, with red indicating higher abundance and blue indicating lower abundance. Hierarchical clustering trees show species and sample similarities; Relative abundance of the rat lower respiratory tract microbiota at the level of phyla **(B)**; relative abundance of rat lower respiratory tract microbiota at the genus level **(C)**. The top 10 phyla/genera are shown.

The abundance clustering heatmap (Figure 5A) visually depicts microbial community abundances. Darker regions indicate higher microbial abundance, while lighter regions signify lower abundance. In Group C, microbial abundance showed a relatively uniform distribution, reflecting the maintenance of typical microbial communities in animals without pollutant exposure. In Group L, microbial abundances were relatively balanced, with minor increases in certain ASVs compared to the control group but minimal overall changes. Group M exhibited greater variability in microbial abundance, with a marked increase in specific ASVs, such as ASV_10 and ASV_7. Group H demonstrated a more concentrated distribution, with significant increases in specific ASVs like ASV_201 and ASV_6. Overall, the microbial communities in the M and H groups underwent significant restructuring, with certain groups showing pronounced increases in abundance while others decreased or maintained low abundance.

At the phylum level (Figure 5B), *Proteobacteria, Firmicutes*, and *Actinobacteriota* were identified as the dominant phyla across all groups. The abundance of *Proteobacteria* significantly increased in Groups L and M but decreased significantly in Group H. In contrast, *Firmicutes* and *Actinobacteriota* exhibited the opposite trend, showing significant decreases in Groups L and M and significant increases in Group H.

At the genus level, the top 10 genera in terms of relative abundance in the lower respiratory microbial community were identified, with the top five genera being *Ralstonia, Pseudomonas, Achromobacter, Burkholderia-Caballeronia-Paraburkholderia*, and *Corynebacterium*.

Across dose groups, the relative abundance of genera exhibited distinct patterns. Between Group C and Groups L and M, a gradual increase in the relative abundance of *Ralstonia* and *Pseudomonas* was observed with increasing exposure doses. Conversely, the relative abundance of *Achromobacter* and *Burkholderia-Caballeronia-Paraburkholderia* showed a decreasing trend. Upon further dose escalation, Group H demonstrated diverse changes in community structure. The relative abundance of *Pseudomonas* increased, while *Ralstonia* decreased. Additionally, less abundant genera, such as *Lactobacillus, Corynebacterium*, and *Atopostipes*, exhibited increased relative abundance, reflecting altered community diversity (Figure 5C).

**Figure 6:**
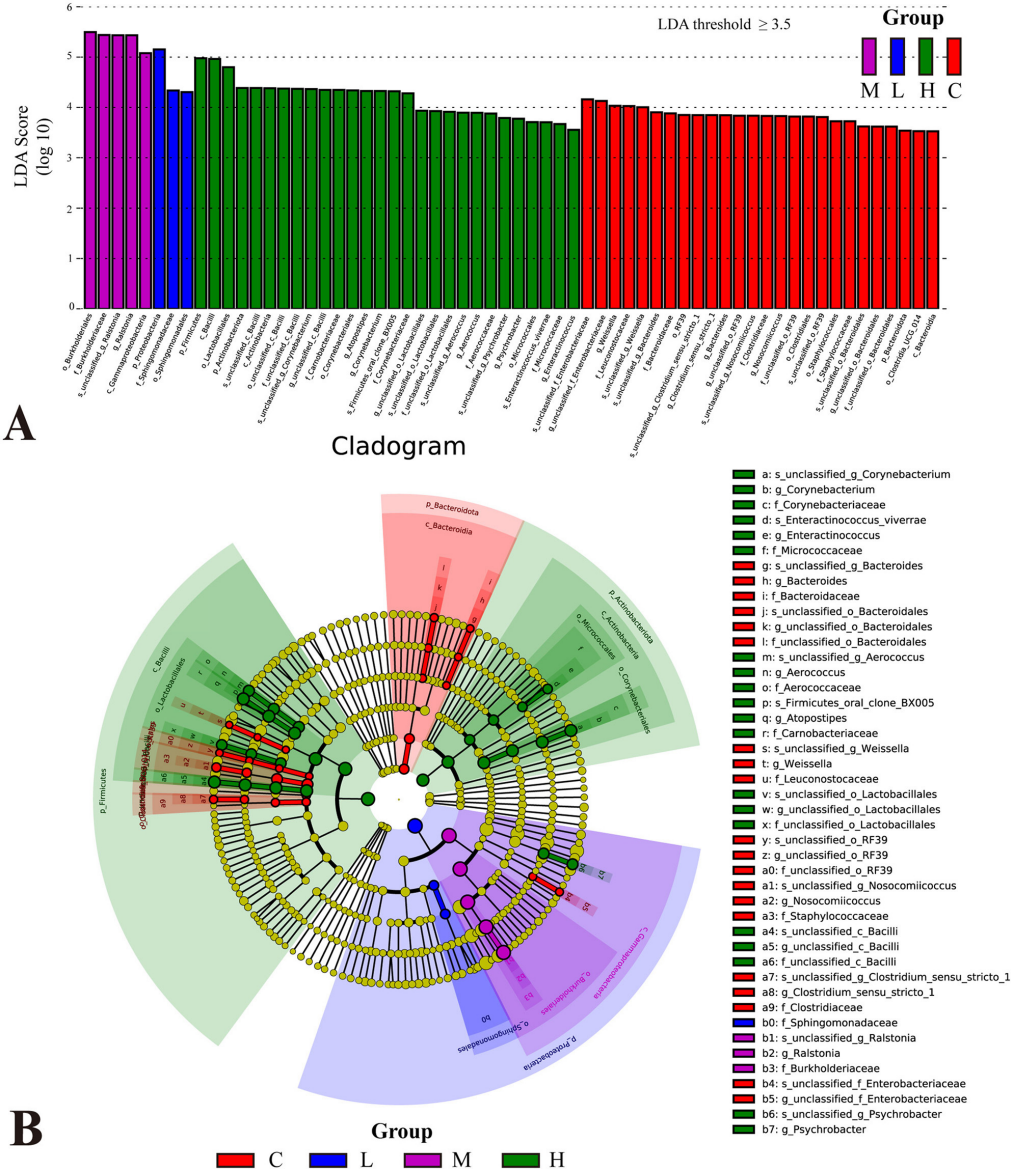
Differential analysis of bacterial flora in the lower respiratory tract of rats based on LEfSe analysis. Histogram of linear discriminant analysis (LDA) scores distribution **(A)**. Taxa with LDA scores >3.5 are considered significantly enriched. Phylogenetic tree illustrating the distribution of significantly different microbial taxa across groups **(B)**. The letters before the underscores represent, in order: phylum(p_), class(c_), order(o_), family(f_), genus(g_), and species(s_).

LEfSe analyses based on relative abundance data were performed to identify bacterial taxa represented by differences between groups. The LDA bar plot results (Figure 6A) revealed significant bacterial taxa associated with each group (LDA threshold ≥ 3.5). In Group C, *Bacteroidota, Clostridiales, Bacteroides, Weissella*, and *Nosocomiicoccus* were significantly enriched, suggesting a dominance of anaerobic and gut-associated taxa. Group L showed increased abundance of *Proteobacteria*, particularly *Sphingomonadales* and *Sphingomonadaceae*. Group M was defined by *Burkholderiales, Burkholderiaceae*, and *Ralstonia*. In contrast, Group H exhibited a broader range of differential taxa, including *Firmicutes, Bacilli, Lactobacillales, Actinobacteriota*, and genera such as *Atopostipes, Aerococcus, Psychrobacter*, and *Enteractinococcus*.

The cladogram (Figure 6B) illustrates the taxonomic distribution of significantly different microbial taxa across groups. The node colors corresponded to the colors in the LDA bar plot, with each color representing significant taxa for a specific group. The central part of the cladogram demonstrated differences among the groups at higher taxonomic levels, such as phylum, class, and order. For example, in Group C, *Bacteroidota*, Clostridiales, and Staphylococcales dominated. However, *Firmicutes* (*Bacilli* and *Lactobacillales*) and *Actinobacteriota* exhibited higher abundance in Group H, while *Proteobacteria* (*Gammaproteobacteria, Burkholderiales, and Sphingomonadales*) were significantly enriched in Group L and Group M, indicating notable variations in the proportions of these major taxa across exposure groups. The peripheral part of the cladogram highlighted differences at lower taxonomic levels, such as genus and species.

### 3.5 Bacterial diversity in alveolar lavage fluid of rats

**Figure 7:**
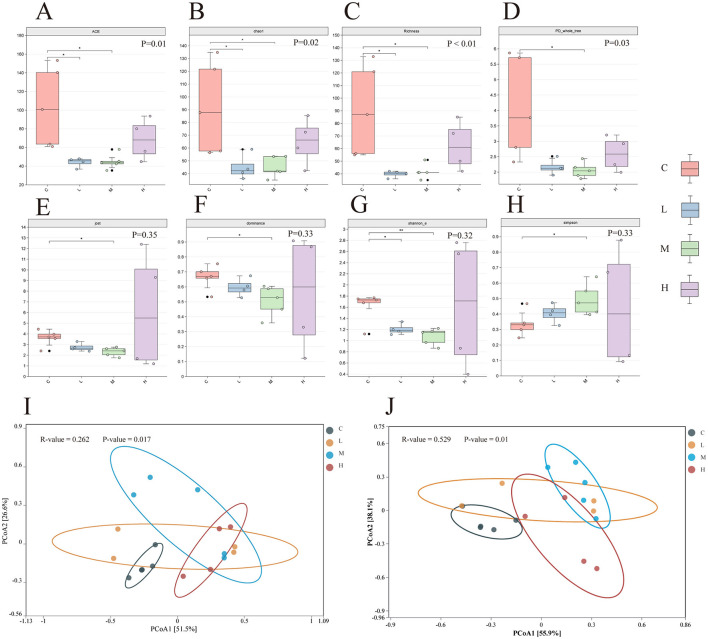
Alpha diversity index of lower respiratory tract microbiota in four groups of rats **(A–H)**. Significance of differences between two groups was determined using the Wilcoxon rank-sum test, while differences among multiple groups were assessed using the Kruskal-Wallis test. *P ≤ 0.05; **P ≤ 0.01. PCoA analysis of lower respiratory tract microbiota in rats under the Bray-Curtis distance algorithm **(I, J)**. ASV level **(I)** and Genus level **(J)**. The ANOSIM (Analysis of Similarities) test was employed to evaluate the significant differences in bacterial β-diversity across multiple groups. C-Group C; L-Group L; M-Group M; H-Group H.

Alpha diversity analysis revealed significant differences in species richness across groups. The Richness, ACE, and Chao1 indices (Figures 7A–C) were significantly higher in Group C compared to Groups L and M (*P* < 0.05 for all), indicating a reduction in microbial richness following metal exposure. Similarly, the PD_Whole_Tree index, reflecting phylogenetic diversity, was significantly lower in the exposure groups relative to Group C (*P* < 0.05), suggesting that inhaled heavy metals decreased phylogenetic breadth (Figure 7D).

In contrast, community evenness and dominance showed no significant group differences. Although the Dominance index suggested a higher proportion of dominant species in Group H and the Jost index indicated slightly greater evenness in this group, neither reached statistical significance (*P* > 0.05; Figures 7E, F).

The Shannon_E and Simpson indices, which account for both richness and evenness, showed a trend of decreasing diversity from Group C to Group H. However, these changes were not statistically significant (Shannon_E: *P* = 0.32; Simpson: *P* = 0.33; Figures 7G, H).

Beta diversity was assessed using Principal Coordinates Analysis (PCoA) based on Bray–Curtis dissimilarity. At the ASV level (Figure 7I), the first two principal coordinates explained 77% of the total variance (PCoA1: 51.5%; PCoA2: 25.5%). ANOSIM results (*R* = 0.262, *P* = 0.017) indicated a moderate but significant separation among the four groups, with Group C microbiota clearly distinct from Group H, while Groups L and M showed partial overlap.

At the genus level (Figure 7J), PCoA1 and PCoA2 explained 55.5% and 38.9% of the variance, respectively, accounting for 94.4% of total variation. Group separation was more pronounced at this taxonomic resolution, as supported by a higher ANOSIM R-value (*R* = 0.529, *P* = 0.01), with clear divergence observed between the control and exposure groups, especially between Group C and Group H.

### 3.6 Correlation of lower respiratory flora with heavy metals and lung function

**Figure 8:**
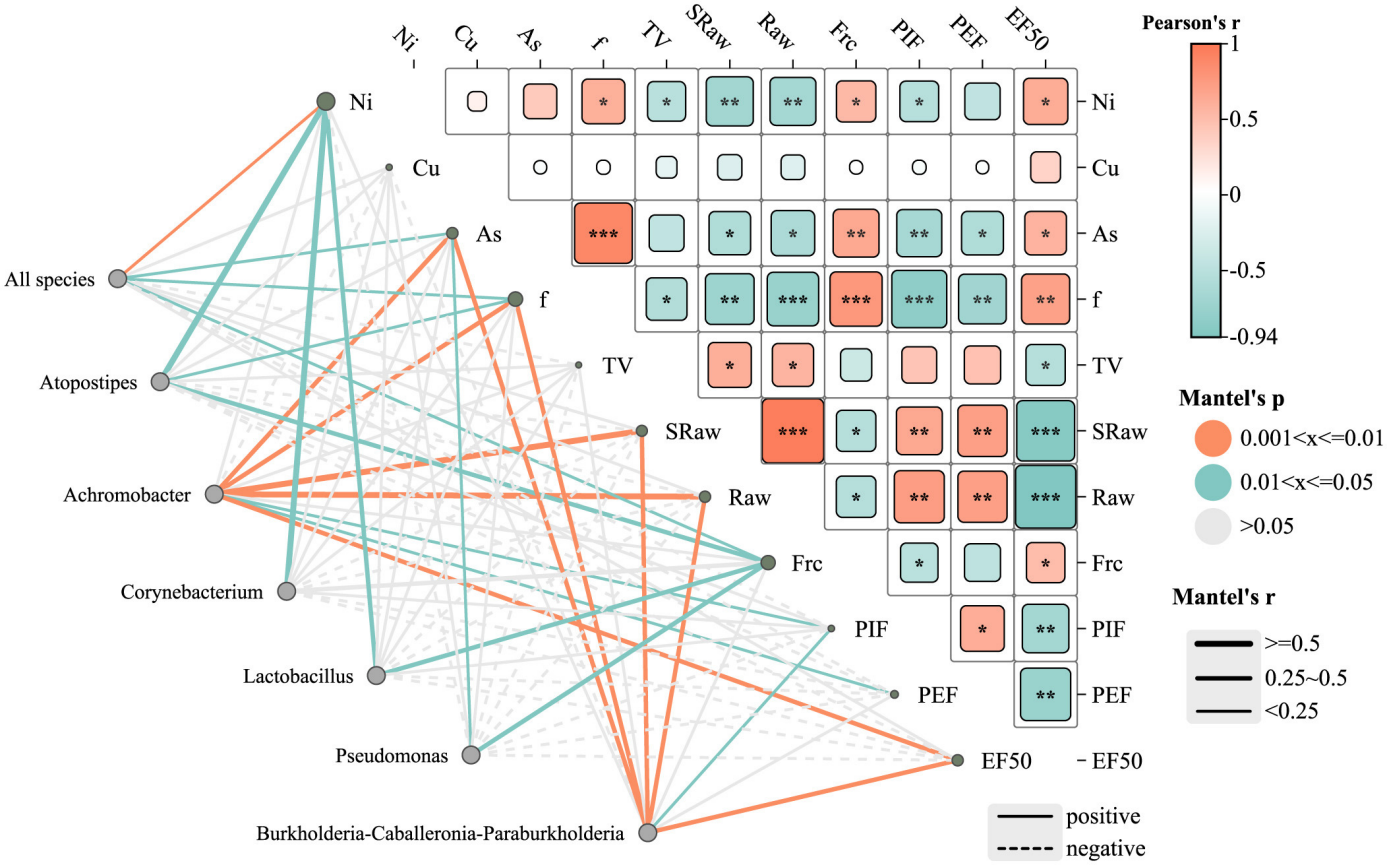
Heat map of the correlation network between lower respiratory tract microbiota, heavy metals, and lung function parameters in rats. *P ≤ 0.05; **P ≤ 0.01; ***P ≤ 0.001. Edge color denotes significance level: green = *P* ≤ 0.05; orange = *P* ≤ 0.01; gray = not significant (*P* > 0.05). Edge style indicates correlation direction: solid = positive, dashed = negative. Edge thickness reflects Mantel correlation strength.

As shown in Figure 8, the network diagram represents microbial genera and environmental/lung function parameters as gray nodes. Green edges indicate significant correlations with moderate strength (0.01 <*P* ≤ 0.05), while orange edges denote stronger significant correlations (0.001 <*P* ≤ 0.01). Gray edges represent statistically non-significant correlations (*P* > 0.05). The type of correlation is further indicated by the line style: solid lines represent significant positive correlations, whereas dashed lines indicate significant negative correlations. The line thickness reflects the strength of the correlation (Mantel's r), with thicker lines indicating stronger correlations.

Several genera, including *Achromobacter, Burkholderia-Caballeronia-Paraburkholderia, Pseudomonas, Lactobacillus*, and *Atopostipes*, were notably correlated with heavy metal concentrations (e.g., Ni and As) and lung function indicators. Among them, *Burkholderia-Caballeronia-Paraburkholderia* exhibited significant positive correlations with EF50 and PEF (*P* ≤ 0.01), suggesting a potential role in preserving lung function under metal exposure. *Pseudomonas* showed a moderate positive correlation with airway resistance (Raw) (*P* ≤ 0.05). In contrast, *Atopostipes* showed a negative correlation with Ni and As exposure, although these associations were not statistically significant (*P* > 0.05), implying a possible sensitivity of this genus to metal stress.

The heatmap further illustrates Pearson correlation coefficients (r) between lung function parameters and metal concentrations. Arsenic (As) levels were negatively associated with multiple lung function indicators, with a significant inverse correlation observed for PEF (^**^P < 0.01), suggesting As exposure may impair airflow capacity. Nickel (Ni) showed strong negative correlations with EF50 and Raw (^***^P < 0.001), indicating substantial impairment of lung function at higher Ni concentrations. Conversely, copper (Cu) exhibited moderate positive correlations with EF50 and PEF (^*^P < 0.05), suggesting a potential stimulatory effect on lung function, possibly through metabolic activation mechanisms.

The “all species” node, representing the overall microbial community, was significantly positively correlated with Ni (*P* ≤ 0.01) and As (*P* ≤ 0.05), suggesting a community-wide response to these metals. No significant correlation was found between Cu levels and the overall community structure. Regarding lung function, the “all species” node showed positive correlations with breathing frequency (f) and functional residual capacity (Frc) (*P* < 0.05), indicating that microbial community composition may influence specific pulmonary parameters.

### 3.7 Functional prediction of respiratory tract communities

Functional gene profiling revealed significant differences among exposure groups. Kruskal–Wallis tests indicated that pathways involved in xenobiotic biodegradation and metabolism varied significantly across groups (*P* < 0.05), whereas core metabolic pathways remained largely conserved (*P* > 0.05).

PICRUSt2 analysis based on KEGG annotations demonstrated that metabolism dominated KEGG Level 1 functions across all groups, accounting for approximately 80%−83% of the total functional capacity. However, a slight reduction in metabolic activity was observed in Group H, suggesting disruption of key microbial functions. Genetic information processing (8%−9%) and cellular processes (~5%) showed mild but consistent dose-dependent alterations.

At the KEGG Level 2 tier, although no pathways reached statistical significance (*P* > 0.05), minor decreases in amino acid metabolism and xenobiotic degradation were noted in the high-dose group. More distinct patterns emerged at KEGG Level 3, where five pathways—including ketone body synthesis, amino acid biosynthesis, and fatty acid degradation—showed significant differences (*P* < 0.05). The low-dose group (L) displayed elevated gene abundances in several pathways, possibly reflecting adaptive compensation, whereas the high-dose group (H) exhibited reduced functional gene expression, indicative of impaired microbial metabolic capacity.

**Figure 9:**
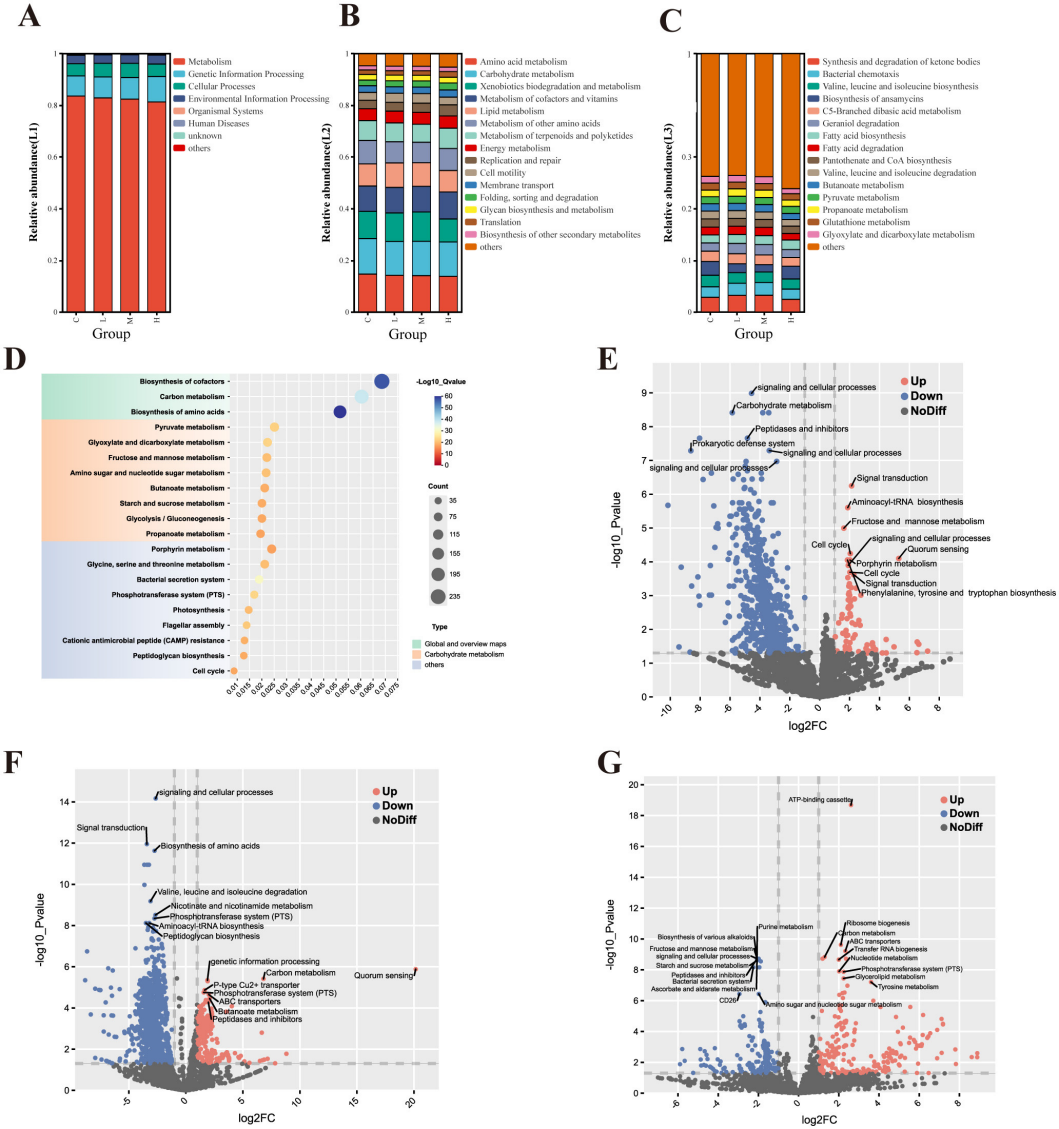
Functional prediction of lower respiratory tract microbiota based on PICRUSt2 analysis with KEGG annotation. Analysis of the pathway composition of the lower respiratory tract microbiota in rats **(A–C)**. Level 1 pathway **(A)**; Level 2 pathway **(B)**; and Level 3 pathway **(C)**. KEGG enrichment analysis of BALF microorganisms in the experimental group and control group **(D)**. KEGG function prediction volcano map of each experimental group compared with the control group **(E-G)**. Group L vs. Group C **(E)**; Group M vs. Group C **(F)**; and Group H vs. Group C **(G)**. Significantly upregulated (red) and downregulated (blue); non-significant (gray).

KEGG enrichment analysis (Figure 9D) highlighted significant upregulation in pathways related to biosynthesis of cofactors, carbon metabolism, and amino acid biosynthesis, with high –log10 (Q-value) scores indicating robust enrichment. Additional enrichment was observed in glycolysis/gluconeogenesis, fructose and mannose metabolism, and amino sugar and nucleotide sugar metabolism, possibly suggesting that heavy metal exposure may trigger metabolic reprogramming to maintain energy homeostasis and structural integrity. Enrichment of the phosphotransferase system (PTS) and bacterial secretion system pathways points to possible microbial adaptation mechanisms involving nutrient uptake and intercellular communication.

Figures 9E–G display volcano plots of KEGG Orthology (KO) terms under different exposure levels. In both low-dose (Figure 9E) and medium-dose (Figure 9F) groups, downregulation was observed in pathways associated with carbohydrate metabolism, signal transduction, and prokaryotic defense systems. Concurrently, upregulation occurred in signal transduction, quorum sensing, and aminoacyl-tRNA biosynthesis pathways, probably suggesting early microbial responses to environmental stress. The medium-dose group showed more pronounced downregulation in amino acid degradation pathways such as valine, leucine, and isoleucine metabolism, as well as in PTS and peptidase-related functions, while upregulation was observed in amino acid biosynthesis and peptidoglycan biosynthesis.

High-dose exposure (Figure 9G) resulted in the most pronounced functional shifts. Notable upregulation was observed in purine metabolism, ATP-binding cassette (ABC) transporters, and ribosome biogenesis pathways, which may reflect enhanced microbial activity associated with protein synthesis and cellular stress responses. In contrast, pathways related to carbohydrate metabolism, amino sugar metabolism, and quorum sensing were significantly downregulated, suggesting potential impairments in core metabolic and communication functions within the microbial community.

### 3.8 Microbial ecological networks analysis of bacterial interactions in BALF

**Figure 10:**
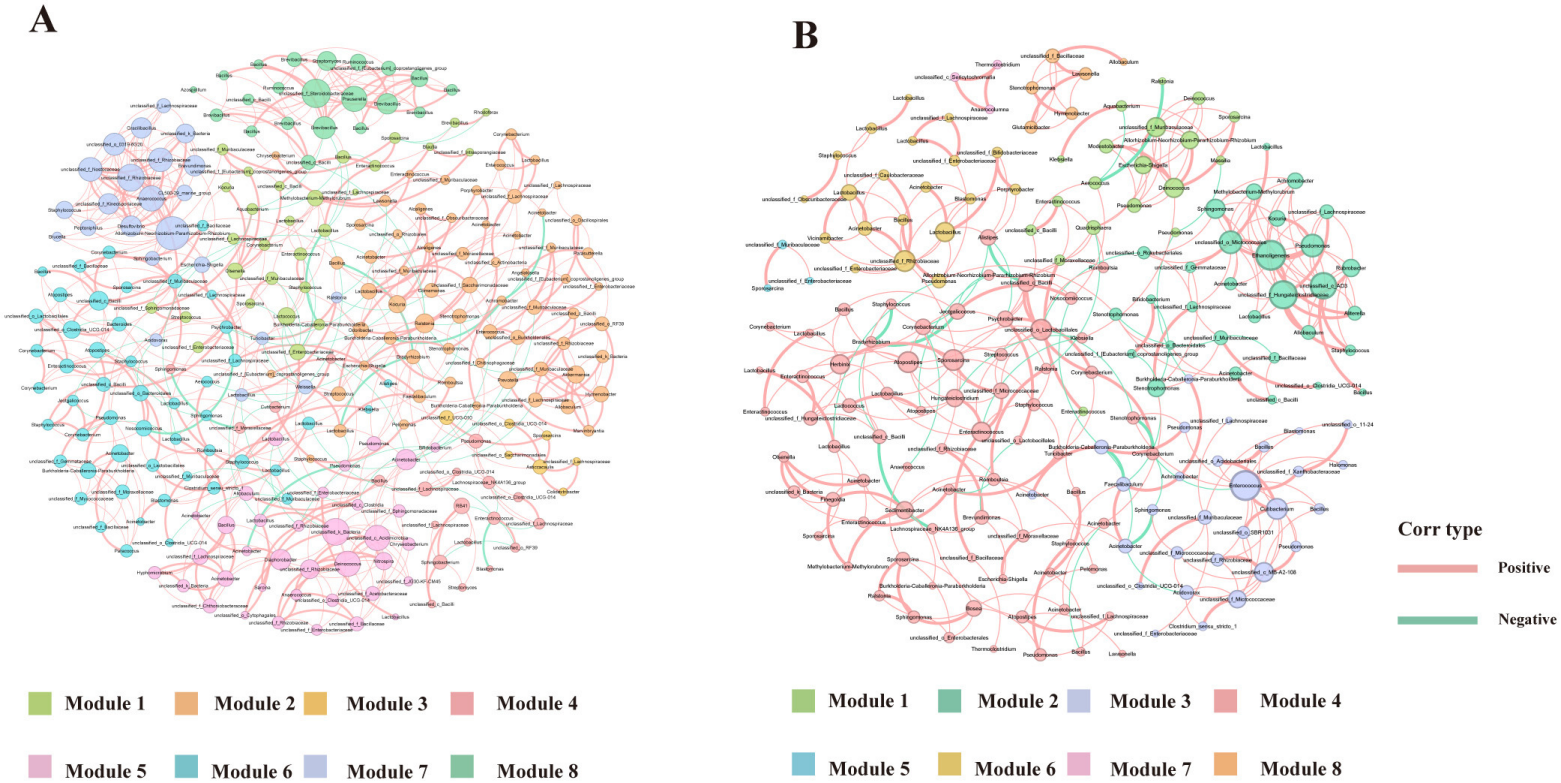
Ecological networks genera illustrating bacterial interactions in the Group Lo **(A)** and Group H **(B)** were constructed based on correlations among bacterial ASVs. Each connection indicates a strong (Spearman's r > 0.6) and significant (*P* < 0.01) correlation. The size of each node is proportional to the number of connections, and the edge thickness is proportional to the weight of each correlation.

To further explore the impact of Ni and As heavy metal concentrations on microbial interactions and relationships, ecological network analyses were conducted by ranking samples based on heavy metal levels. The samples were divided into low-dose (Lo, Figure 10A) and high-dose (Hi, Figure 10B) groups at the 50% threshold. Nodes in the networks were annotated with their corresponding phyla.

Network complexity differed significantly between the two groups. Compared to the Lo group, the Hi group exhibited a less complex network, characterized by fewer edges (367 vs. 521), fewer nodes (184 vs. 251), a lower average degree (3.989 vs. 4.719), and reduced modularity (0.712 vs. 0.823) (Table 4). Keystone genera also differed between the groups. In the Hi group, *Atopostipes* and *Sporosarcina* were identified as keystone taxa, whereas in the Lo group, the keystones included *Bacill, Gammaproteobacteria, Alphaproteobacteria*, and *Acidimicrobiia*. While direct links between these genera and lung function or damage are limited, the differences in keystone taxa suggest notable shifts in microbial interactions between low-dose and high-dose exposure groups.

**Table 4:** Topological properties of networks for the two groups.

**Group**	**Nodes**	**Edges**	**Modularity (MD)**	**Clustering coefficient (CC)**	**Average path length (APL)**	**Network diameter (ND)**	**Average degree (AD)**
Lo	250	641	0.748	0.46	4.984	10	5.128
Hi	184	367	0.712	0.45	5.697	13	3.989

Further analysis focused on the largest module in each network, defined by the highest number of nodes. In the Lo group, Module 2 represented the largest module (25.6% of the network), while in the Hi group, Module 3 dominated (39.67%). The dominant genera in these modules varied as well, with the Lo group primarily comprising genera such as *Ralstonia, Lactobacillus*, and *Prevotella*, while the other group was predominantly composed of genera like *Atopostipes, Enteractinococcus*, and *Sporosarcina*.

The network diagrams reveal that most relationships between microbial taxa were positive, with only a small fraction displaying negative correlations. Notably, negative relationships involved genera such as *Lactobacillus* and *Burkholderia-Caballeronia-Paraburkholderia* in their interactions with other taxa. These changes in modularity and relationships suggest a reorganization of microbial network structures under heavy metal exposure, potentially reflecting the effects of environmental stress on community functionality.

## 4 Discussion

This study investigated, for the first time, the effects of mixed aerosol exposure to nickel (Ni), copper (Cu), and arsenic (As) on rat lungs using high-throughput sequencing technology, and analyzed the correlation with the respiratory microbiota. Although the effects of combined heavy metal exposure on lung tissues are an emerging field in air pollution research, the underlying mechanisms remain unclear. The findings demonstrated that sub-chronic combined exposure to mixed aerosols of Ni, Cu, and As resulted in significant lung damage and reduced lung function in rats. Additionally, combined exposure to these heavy metals altered the composition of the respiratory microbiota. Importantly, a significant association was found between changes in several respiratory bacterial communities and decreased lung function. Overall, this study reveals the potential role of respiratory microbiota in lung function reduction induced by combined heavy metal exposure and provides new insights into the mechanisms underlying heavy metal-induced lung function impairment.

Exposure to heavy metals may lead to reduced lung function (Wu et al., 2022). After exposure to a mixture of three heavy metals, rats in the M and H groups exhibited significant inflammatory cell infiltration, thickening of alveolar walls, and deformation of alveoli. These pulmonary injuries may impair airway patency and affect lung ventilation function. Compared to the control group, the airway resistance indicators (sRaw and Raw) in the heavy metal exposure groups were significantly elevated. A reduction in tidal volume can trigger a compensatory increase in respiratory frequency in rats to maintain adequate ventilation (Liu, 2022), consistent with our results. With escalating levels of heavy metal exposure, compensatory pulmonary overexpansion was observed, as indicated by a significant increase in functional residual capacity (FRC) in the H group. FRC serves as an indicator of lung overinflation (Laveneziana and Palange, 2012; Aalstad et al., 2018); The PEF values in the exposure groups were all lower than those in the control group, indicating that combined heavy metal exposure exacerbates airway obstruction. Peak inspiratory flow (PIF), which is influenced primarily by respiratory muscle strength and airway patency, was also reduced in exposed rats. This decline may be attributed to weakened respiratory muscle function (Liu, 2022). Changes in muscle strength and endurance should be interpreted dependent on the density of slow muscle fibers (Fuso et al., 2012). Notably, microvascular lesions primarily affect slow muscle fibers, thereby impacting endurance. Our study shows that PIF decreases with increasing heavy metal concentration, indicating that the decline in lung function in rats due to heavy metals may be related not only to increased airway resistance but also to weakened respiratory muscle strength and complications in lung microvasculature.

High-throughput sequencing confirmed sufficient data depth. At the phylum level, Groups L and M showed increased *Proteobacteria* and decreased *Firmicutes* and *Actinobacteria*, consistent with findings in PM_2.5_ and ventilation-exposed animal models, and patients with respiratory diseases like asthma and COPD (Hilty et al., 2010; Chen, 2022). In contrast, Group H had lower *Proteobacteria* and higher *Firmicutes*, suggesting that high heavy metal levels may suppress sensitive taxa while favoring resistant ones. This pattern aligns with studies reporting *Proteobacteria* decline and *Firmicutes* enrichment under heavy metal stress (Duan et al., 2024; Jin et al., 2024). Increased *Firmicutes* in Group H may reflect ecological adaptation or selective pressure. Minor phyla remained stable, indicating limited responsiveness.

At the genus level, *Ralstonia, Pseudomonas*, and *Achromobacter* dominated. In Group H, *Pseudomonas* increased while *Ralstonia* declined. Rare genera such as *Lactobacillus, Corynebacterium*, and *Aerococcus* also increased, possibly indicating adaptive or protective roles under metal stress. Then, the high relative abundance of Group H for *Pseudomonas* may reflect selective environmental pressure. If certain metals or pollutants favor *Pseudomonas* growth and inhibit *Ralstonia, Pseudomonas'* relative abundance would rise (Choudhary and Sar, 2016). This could be due to *Pseudomonas'* stronger adaptability and more efficient metal-resistance mechanisms (Adhikary et al., 2019).

Alpha diversity indices (ACE, Chao1, PD) showed significant reductions in richness and phylogenetic diversity in exposure groups, especially under high-dose exposure, in line with previous research on cadmium and air pollution (Tao et al., 2020; Laiman et al., 2022). Shannon_E and Simpson indices indicated lower diversity and evenness, with a dose-dependent decline. However, changes in dominance were not statistically significant, though trends pointed to enrichment of resistant genera like *Burkholderia* and *Pseudomonas* (Awais et al., 2024). Beta diversity (PCoA) showed clear group separation. The high-dose group had the greatest dispersion, indicating disrupted microbial stability and increased heterogeneity, likely due to selective pressure favoring metal-resistant taxa and suppressing sensitive ones. Genus-level resolution revealed stronger group differences than ASV-level, highlighting taxonomic restructuring. These results echo NMDS-based studies on polluted soils showing increased heterogeneity under heavy metal stress (Wen et al., 2024).

The LDA analysis (Figure 6A) revealed dose-dependent community shifts. In Group C, *Bacteroidota* and *Bacilli* (e.g., *Weissella*) predominated, consistent with a stable, anaerobe-dominated community, suggesting a relatively stable microbiota. Low-dose exposure caused minimal structural changes, while medium-dose exposure enriched resistant taxa like *Proteobacteria*. In the high-dose group, enrichment broadened to include multiple phyla (*Firmicutes, Actinobacteriota*) and opportunistic genera such as *Atopostipes, Aerococcus*, and *Psychrobacter*, suggesting a possible shift toward dysbiosis and colonization by opportunistic or environmental bacteria. The cladogram (Figure 6B) further confirmed phylogenetic divergence across groups, highlighting the distinct microbial signatures induced by increasing metal burden. These patterns align with previous findings that heavy metal exposure can reduce microbial diversity, disrupt metabolic function, and promote inflammatory responses (Giambò et al., 2021). Consistent with our observations, study reported that rising Ni, Cu, and As sharply lower α-diversity and favor metal-tolerant generalists (e.g., Gammaproteobacteria, Bacillus) as keystone generalists, simplifying microbial networks under heavy-metal stress (Qi et al., 2022). Together, these findings suggest that heavy metal exposure-associated alterations in the lower airway microbiome might contribute to compromised mucosal immunity and increased susceptibility to lung injury.

The Mantel Test (Figure 8) provided critical insights into the relationships between microbial genera, environmental factors, and lung function parameters, highlighting the complex interplay between microbiota and heavy metal exposure. Genera such as *Achromobacter, Burkholderia-Caballeronia-Paraburkholderia*, and *Pseudomonas* were significantly positively correlated with heavy metal concentrations like Ni and As. Notably, *Burkholderia* was linked to better respiratory parameters, possibly due to its robust oxidative stress defenses and detoxification systems (Eissa, 2024), which have been shown to enhance bacterial survival and support host adaptability. *Pseudomonas* was moderately correlated with Raw, reflecting its adaptive capacity in polluted environments. Conversely, *Atopostipes* showed a non-significant negative correlation with Ni and As, hinting at suppression under metal stress, aligning with reports of metal-induced microbial imbalance and inflammation (Popov Aleksandrov et al., 2021). However, while *Pseudomonas* may help maintain ecological stability, its pathogenic potential, particularly in chronic lung diseases such as COPD, cannot be overlooked (Jingsheng et al., 2023). Correlation analysis with the three heavy metals Ni, Cu, and As revealed that *Lactobacillus, Corynebacterium*, and *Atopostipes* were positively correlated with Ni, while *Achromobacter* and *Pseudomonas* were positively correlated with As (Figure 8). *Lactobacillus*, known for its immunomodulatory effects (Huang et al., 2014), may support lung health through the gut-lung axis (Heeney et al., 2018). In contrast, *Corynebacterium* and *Actinobacteria*—frequently found in respiratory infections—may indicate potential pathogenicity under environmental stress (Sze et al., 2015; Li H. et al., 2019).

Functional prediction using PICRUSt2 (Figures 9A–C) revealed that core metabolic pathways, including carbohydrate metabolism, amino acid metabolism, and lipid metabolism, were largely conserved across all groups (*P* > 0.05). Heavy metal exposure can impair the metabolic potential of microbial communities, thereby disrupting host nutrient absorption, energy metabolism, and respiratory health (He et al., 2020; Liu et al., 2024). In our study, compositional differences in the BALF microbiota across exposure groups suggested that microbial community structure is sensitive to heavy metal stress, necessitating further investigation into functional gene diversity.

The KEGG enrichment analysis (Figure 9D) demonstrates a significant perturbation in metabolic pathways, particularly those associated with carbohydrate metabolism, amino acid biosynthesis, and energy production processes. The most significantly enriched pathways included biosynthesis of amino acids, carbon metabolism, and pyruvate metabolism, aligning with previous studies that indicate microbial communities respond to heavy metal stress by enhancing essential metabolic pathways to maintain cellular stability and energy homeostasis (Prabhakaran et al., 2016; Ayangbenro and Babalola, 2017). The enrichment of pathways related to bacterial secretion systems and cationic antimicrobial peptide (cAMP) resistance suggests that microbial communities are actively deploying defense mechanisms against environmental stress, particularly under toxic metal exposure conditions (Patil et al., 2024).

In the differential expression analysis (Figures 9E–G), significant differences were observed across experimental groups. Low-dose exposure predominantly affected genes involved in signal transduction, prokaryotic defense systems, and carbohydrate metabolism. At medium doses, genes related to amino acid biosynthesis, branched-chain amino acid degradation (valine, leucine, isoleucine), and membrane transport via the phosphotransferase system (PTS) were enriched. In contrast, high-dose exposure triggered a pronounced microbial stress response, with upregulation of purine metabolism, ABC transporters, and ribosome biogenesis, indicating a shift toward protein synthesis and cellular repair mechanisms, typical of environmental stress adaptation. These findings align with previous reports that metals such as mercury (Hg), arsenic (As), and cadmium (Cd) can activate detoxification pathways, where metal-glutathione complexes are expelled through ABC transporters to alleviate toxicity (Pearson and Cowan, 2021). Study demonstrated that microbial systems often trade off metabolic efficiency for stress survival, reallocating resources toward protective pathways under adverse conditions (Escoll and Buchrieser, 2019). Such adaptive responses may have broader implications for microbial ecology and environmental resilience.

Ecological network analysis revealed that microbial interactions in the high-dose exposure group (Hi) were significantly simplified, as evidenced by a reduction in network nodes and edges. This suggests that high concentrations of nickel and arsenic suppress sensitive microbial taxa, thereby reducing microbial diversity and weakening overall community connectivity. Such network simplification is indicative of ecological stress and aligns with prior findings showing that heavy metal exposure exerts strong selective pressure, favoring metal-tolerant species while inhibiting sensitive groups. Similar patterns have been observed in other environmental studies, where pollutants such as polycyclic aromatic hydrocarbons (PAHs) and heavy metals led to reduced microbial network complexity and stability, ultimately making ecosystems more vulnerable (Wang et al., 2024). These changes underscore a shift in community structure, with cooperative interactions being replaced by competitive or stress-adaptive dynamics. For instance, the decline of sensitive genera like *Acidobacteria* and *Verrucomicrobia* has been previously reported under heavy metal stress, contributing to lower modularity and increased inter-species competition (Ma et al., 2020). In our study, this trend is further reflected in the shift of keystone genera from *Lactobacillus* and *Ralstonia* in the low-dose group to stress-adaptive genera such as *Atopostipes* and *Sporosarcina* in the high-dose group. These findings highlight how heavy metal exposure not only simplifies microbial networks but also reorganizes keystone taxa, potentially altering key ecological functions such as nutrient cycling and immune regulation.

This study also has several limitations that warrant consideration. First, the lack of single-metal exposure groups prevents the differentiation of independent effects and interactions among the heavy metals in mixed exposure. Future studies should include single-metal exposure groups to clarify these contributions and provide more comprehensive insights. Second, Sample sizes (*n* = 5 for each group) may limit the generalizability of our conclusions, we plan to increase sample sizes in future experiments to enhance the robustness and reproducibility of our results. Third, while 16S rDNA sequencing offered valuable functional predictions of the lower respiratory microbiota, the absence of direct validation through metabolomics, transcriptomics, or functional assays limits the ability to confirm the predicted microbial functions and their relevance to host physiology. Incorporating high-throughput techniques and metabolite detection in future studies could enhance the understanding of microbial functional changes under heavy metal stress. Additionally, the study relies on endpoint data without capturing the temporal progression of microbial dysbiosis and its link to lung injury, highlighting the need for longitudinal analyses.

## 5 Implications and future perspectives of this study

This study reveals that exposure to heavy metals such as Ni, Cu, and As induces specific disruptions in the respiratory microbiota of rat BALF, including the enrichment of *Pseudomonas* and *Burkholderia* and depletion of *Ralstonia*, which may act as early biomarkers of dysbiosis linked to lung dysfunction. Unlike previous work focused on gut microbiota or general respiratory inflammation, our study highlights for the first time the distinct compositional and predicted functional alterations of the lower-airway microbiota under co-exposure to multiple metals. Although based on 16S rRNA data, our findings provide new insights into potential microbial symbioses and their association with host injury, offering a basis for hypothesis-driven mechanistic research. Future studies applying multi-omics and longitudinal models are essential to establish causality and delineate microbial contributions to lung injury. These results also support the integration of microbiota-sensitive endpoints into air pollution toxicology and regulatory frameworks, emphasizing the need for strengthened emission controls, metal-specific PM_2_.5 monitoring, and early-warning strategies tailored to vulnerable populations.

## 6 Conclusion

This study demonstrates that mixed exposure to nickel (Ni), copper (Cu), and arsenic (As) induces significant, dose-dependent lung injury and alters the composition and diversity of the lower respiratory tract microbiota in rats. The exposure led to microbial dysbiosis, characterized by enrichment of metal-tolerant genera such as *Pseudomonas* and *Burkholderia*, and depletion of sensitive taxa including *Ralstonia* and *Actinobacteria*. These microbial changes were closely associated with impaired lung function, suggesting a potential mechanistic link. Ecological network analysis further revealed simplified microbial interactions and a shift in keystone taxa under high-dose exposure. Although the exact mechanisms remain to be clarified, these findings highlight the impact of heavy metal exposure on respiratory microecology and support the need for stricter environmental control, particularly of Ni and As emissions, as well as further studies to explore microbiota-based strategies for mitigating lung injury.

## Data Availability

The datasets presented in this study can be found in online repositories. The names of the repository/repositories and accession number(s) can be found below: https://www.ncbi.nlm.nih.gov/, PRJNA1253313.

## Footnotes

^1^ https://github.com/vegandevs/vegan

^2^ https://github.com/Hy4m/linkET

^3^ https://gephi.org/

^4^ https://www.chiplot.online/
