# Supplementary material for: Effects of combined exposure to heavy metals on lower respiratory flora and its role of lung injury in rats
Source: Front Microbiol. 2025 Jun 13;16:1615130. doi: 10.3389/fmicb.2025.1615130 (PMC12202419; doi:10.3389/fmicb.2025.1615130)
Supplement: Supplementary file 1 [file Table_1.docx]

Supplementary Material

Table

**Supplementary Table 1.** This table summarizes all abbreviations used throughout the manuscript, including chemical elements, lung function indices, microbial diversity metrics, computational tools, and statistical terms. Abbreviations are listed in the order of their first appearance and categorized by research context for clarity.

| **Abbreviation** | **Full Term** |
| --- | --- |
| Ni | Nickel |
| Cu | Copper |
| As | Arsenic |
| SD | Sprague–Dawley (rat strain) |
| SPF | Specific Pathogen-Free |
| PBS | Phosphate-Buffered Saline |
| H&E | Hematoxylin and Eosin |
| f | Respiratory Frequency |
| Tv | Tidal Volume |
| sRaw | Specific Airway Resistance |
| Raw | Airway Resistance |
| PIF | Peak Inspiratory Flow |
| PEF | Peak Expiratory Flow |
| FRC | Functional Residual Capacity |
| EF50 | Expiratory Flow at 50% Tidal Volume |
| BALF | Bronchoalveolar Lavage Fluid |
| 16S rDNA | 16S Ribosomal DNA |
| ASV | Amplicon Sequence Variant |
| ACE | Abundance-based Coverage Estimator |
| Chao1 | Chao1 Richness Estimator |
| PD_whole_tree | Phylogenetic Diversity Whole Tree |
| Shannon_E | Shannon Evenness Index |
| Simpson | Simpson Diversity Index |
| Richness | Species Richness Index |
| Jost | Jost's Diversity Index |
| Dominance | Dominance Index |
| QIIME2 | Quantitative Insights Into Microbial Ecology v2 |
| PICRUSt2 | Phylogenetic Investigation of Communities by Reconstruction of Unobserved States 2 |
| KEGG | Kyoto Encyclopedia of Genes and Genomes |
| KO | KEGG Orthology |
| PTS | Phosphotransferase System |
| DESeq2 | Differential Expression Sequencing 2 |
| fastp | An all-in-one FASTQ preprocessing tool |
| FLASH | Fast Length Adjustment of Short Reads |
| PCoA | Principal Coordinates Analysis |
| ANOSIM | Analysis of Similarities |
| LEfSe | Linear Discriminant Analysis Effect Size |
| LDA | Linear Discriminant Analysis |
| iNAP | Integrated Network Analysis Pipeline |
| MEN | Molecular Ecological Network |
| MB | Meinschausen-Bühlmann |
| SPEIC-EASI | Sparse Inverse Covariance Estimation for Ecological Association Inference |
| APL | Average Path Length |
| CC | Clustering Coefficient |
| ND | Network Diameter |
| AD | Average Degree |
| MD | Modularity |
| R | R Programming Language |
| SPSS | Statistical Package for the Social Sciences |
